# Development of in vitro cardiovascular tissue models within capillary circuit microfluidic devices fabricated with 3D stereolithography printing

**DOI:** 10.1007/s42452-023-05459-9

**Published:** 2023-08-19

**Authors:** Aibhlin Esparza, Nicole Jimenez, Binata Joddar, Sylvia Natividad-Diaz

**Affiliations:** 1 Department of Metallurgical, Materials, and Biomedical Engineering (MMBME), The University of Texas at El Paso (UTEP), El Paso, TX, USA.; 2 3D Printed Microphysiological Systems Laboratory, The University of Texas at El Paso, El Paso, TX, USA.; 3 Inspired Materials & Stem-Cell Based Tissue Engineering Laboratory (IMSTEL), The University of Texas at El Paso, El Paso, TX, USA.; 4 Border Biomedical Research Center, University of Texas at El Paso, El Paso, TX, USA.

**Keywords:** Microfluidic device, 3D tissue model, Cardiac, Microenvironment

## Abstract

This study presents the development and morphology analysis of bioinspired 3D cardiovascular tissue models cultured within a dynamic capillary circuit microfluidic device. This study is significant because our in vitro 3D cardiovascular tissue models retained within a capillary circuit microfluidic device provide a less expensive, more controlled, and reproducible platform for more physiologically-relevant evaluation of cellular response to microenvironmental stimuli. The overall aim of our study is to demonstrate our cardiovascular tissue model (CTM) and vascular tissue model (VTM) actively changed their cellular morphology and exhibited structural reorganization in response to biophysical stimuli provided by microposts within the device tissue culture chambers during a 5-day period. The microfluidic device in this study was designed with the Young–Laplace and Navier–Stokes principles of capillary driven fluid flow and fabricated with 3D stereolithography (SLA) printing. The cardiac tissue model and vascular tissue model presented in this study were developed by encapsulating AC16 cardiomyocytes (CTM) and Human umbilical vein endothelial cells (VTM) in a fibrin hydrogel which were subsequently loaded into a capillary circuit microfluidic device. The cardiovascular tissue models were analyzed with fluorescent microscopy for morphological differences, average tube length, and cell orientation. We determined the VTM displayed capillary-like tube formation and the cells within both cardiovascular tissue models continued to elongate around microposts by day-5 which indicates the microfluidic system provided biophysical cues to guide cell structure and direction-specific organization.

## 1 Introduction

Human heart tissues and cardiovascular diseases are a challenge to study for novel drug discovery and fundamental cellular/molecular processes due to the limited availability of physiologically-relevant models in vitro [1–3]. While animal models have been used to study heart structure, previous research has demonstrated notable differences from human cardiac and vascular physiology including vascular flow rate, biochemical signaling, and gene expression [4–6]. In vitro 3D tissue models with microfluidic culture systems provide an inexpensive, more controlled, and reproducible platform for better quantification and evaluation of cellular processes exposed to biochemical or biophysical stimulus [4, 7–10]. Incorporating human cardiovascular cells into microfluidic devices provides a novel physiologically-relevant system to study biochemical and biophysical cellular responses and processes within cardiovascular tissue in vitro [11–15].

Current 3D cardiovascular tissue models incorporate several types of human cardiovascular lineage cells including those derived from human induced pluripotent stem cells (hiPSCs) [2, 16–18]. However, many of these systems necessitate more physiologically-relevant microenvironments that provide dynamic fluid circulation and biophysical cues to guide cellular structural organization [2, 19, 20]. With the integration of 3D stereolithography printing to manufacture microfluidic devices, microenvironments can be designed and tailored to the types of cells and tissues of interest, including channels, micropillars, microposts, inlets, and outlets for in vitro studies. By doing so, it allows for detailed studies of cell-extracellular matrix (ECM) interactions that are critical to the development and physiological functions for tissues [21–23]. Moreover, these customized microenvironments promote more stable capillary-like tube formation within 3D cardiovascular tissue models including the geometries, branching structures, substrate material, and surfaces [24]. Specifically, microfluidic devices provide a versatile, reproducible microenvironment to study the effects of biophysical signals on 3D tissues in vitro [25].

Fabrication techniques to make microfluidic devices include photolithography, casting, injection molding and 3D printing. Photolithography incorporates polydimethylsiloxane (PDMS), where SU-8 is used to create a master mold for PDMS microfluidic devices. However, PDMS requires multiple steps and processes to create a final microfluidic device and can be time consuming in initial steps [26, 27]. Moreover, 3D printing has become an inexpensive, fast, and reproducible manufacturing technique. This technique gives the user the ability to print devices in a single process. 3D Stereolithography (SLA) printing operates on the principle of high-energy laser that harden liquid resin layer by layer create a solid shape. Microfluidic devices can be 3D printed and tested with different types of cells and microenvironments to study specific interactions [28, 29].

Moreno-Rivas et al. create biocompatible microfluidic devices with stereolithography (SLA) printing using 3 different types of resin, Clear, High Temp, and Dental Resin LT [30]. Microfluidic devices were designed with rectangular microchannels, printed using a Form 2 Formlabs printer, and surface treated with Poly-d-Lysine before culturing HeLa cells for a 5 day study. Clear and High Temp resins were recommended to ensure reproducibility of the printed devices, however, the High Temp resin affected the survival of the cells, indicating a potential toxic effect. This toxic effect was not demonstrated in Dental Resin LT or Clear resin, where these resins did not affect cell viability, indicating a longer incubation time would be better to describe the biocompatibility of each resin.

Salmon et al. describes a 3D printed platform aimed to work with an organoid system and provides a way to vascularize organoids [31]. Using a FormLabs2 printer, the microfluidic chip design included an “open well” to allow for easy and direct accessibility to the compartment with the organoid, as well as, a central organoid chamber that was capable of seeding vascular cells through an inlet. Human induced pluripotent stem cell (hiPSC)-derived endothelial cells and pericytes were added into the microfluidic chips over the course of 25 days and resulted in angiogenic sprouting and network formation, as well as active perfusion and vascularization. Additionally, to supporting the organoid and vasculature, 3D printing technology can create biocompatible, long-term culture, and adaptability for devices conformable to co-culture of different cell types and vascularization.

Veldhuizen et al. demonstrates a microfluidic device designed to co-culture three cell types and promote anisotropy of resultant cardiac tissues [16]. hiPSC-derived cardiomyocytes (CMs) were cocultured with human cardiofibroblasts (CFs), encapsulated within the hydrogel, and placed into the device with staggered microposts to form a 3D tissue. Co-cultured tissues were allowed to grow up to 3 weeks and tissues formed in the device displayed mature cellular organization, production of proteins, and upregulation of genes, where microposts served as microenvironment cues that induced cell lengthening and alignment observed in human myocardium. Tissue function increased with synchronicity of spontaneous beating and calcium transients.

**Fig. 1:**
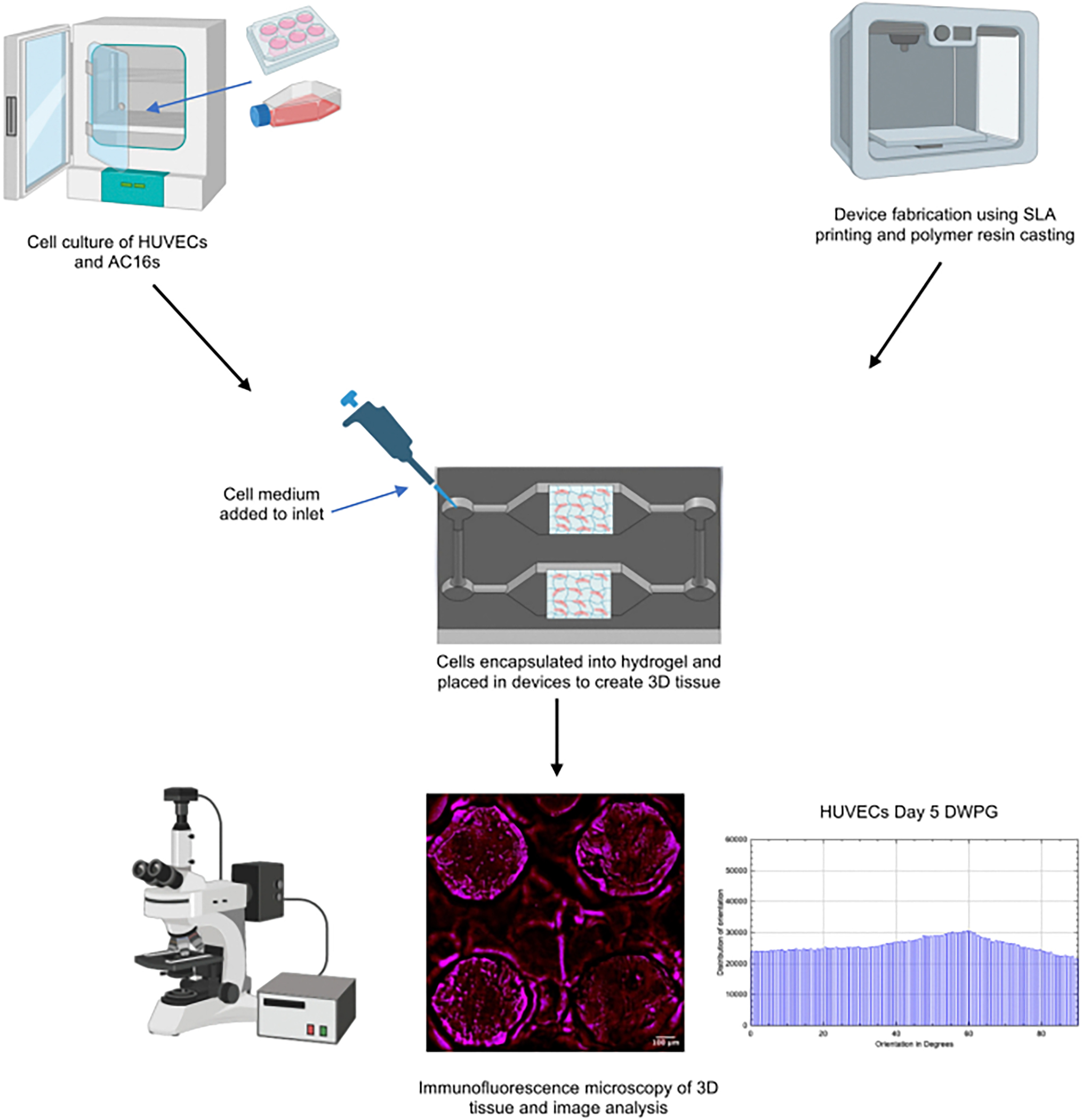
Work flow implemented in this study including: microfluidic device design and fabrication, 3D Cardiovascular tissue model development, and sample characterization with fluorescence microscopy and computational image analysis

Capillary circuit microfluidic devices function without the help of external pumps, valves, or support, and liquid movement is driven by capillary forces determined by the geometry and surface chemistry of microchannels through the microfluidic device [32, 33]. Our study presents a novel 3-step method and capillary driven-flow microfluidic platform to develop mechanically-responsive 3D cardiovascular tissue models in vitro. The method implemented in this study consisted of three major steps: (1) Microfluidic device design, manufacture, and validation, (2) 3D Cardiovascular tissue model development, and (3) Sample characterization with fluorescence microscopy and computational image analysis (Fig. 1). The capillary-flow microfluidic device presented in this study was manufactured with a 3D stereolithography (SLA) printed mold and is a closed circuit system that operates on the principles of capillary action which allows continuous fluid movement without the need for external power supply. Human umbilical vein endothelial cells (HUVECs) and human cardiomyocytes (AC16s) were encapsulated into a fibrin hydrogel and cultured in the microfluidic devices for 1, 3, and 5 days. Fibrin was used in this study since it is a naturally occurring protein in the human body and an essential blood clotting component [34]. Fibrin has been used as an extracellular matrix product for cardiovascular cell encapsulation due to its ability to provide many cellular adhesion molecules and supports growth of cardiovascular cells in vitro [35]. Several studies have demonstrated fibrin hydrogels may be used to study cell behavior, cellular alignment, cardiomyocyte beating, vessel formation, wound healing, and function of 3D engineered cardiovascular tissues [34, 35]. In this study, the vascular tissue models and cardiac tissue models demonstrated statistically significant differences in cell alignment and cell orientation in samples housed in devices with microposts (Devices with posts, grid, DWPG) relative to devices without microposts (Device without posts, DWoP) and standard transwell inserts.

In the next section we describe the design to fabrication process of the microfluidic devices, encapsulation of HUVECs and AC16s into fibrin hydrogels, and characterization and analysis of vascular and cardiac tissue models. Section 3 demonstrates the ability of the microfluidic system to maintain capillary fluid flow, guide cell alignment, and vascular network formation within both tissue models. In Sect. 4, we discuss important findings of specific cell alignment and structural organization within our cardiovascular tissue models over the course of 5 days. Finally, Sect. 5 describes the relevance and limitations of this study along with future directions for our microfluidic system.

## 2 Methods

### 2.1 Microfluidic device design, fabrication, and validation

#### 2.1.1 Capillary driven-flow microfluidic device design

The capillary driven-flow microfluidic device presented in this study was manufactured with a 3D stereolithography (SLA) printing and polymer casting method. The device is a closed circuit system that operates on the principles of capillary action which allows continuous fluid movement without the need for external power supply. In these studies, the device remained open, while inside a petri dish; however, a lid can easily be designed to encase the entire device. Microfluidic capillary pumps incorporate different geometries of microstructures and surface properties to generate capillary pressure and self-regulated liquid delivery [32]. Our capillary-flow microfluidic device was designed in Solidworks CAD Software (Solidworks Corporation) with the Young–LaPlace and Navier–Stokes equations for capillary fluid flow with dimensional constraints dictated by the printing resolution of a Formlabs Form3B SLA printer and Formlabs V4 Clear Resin.

**Fig. 2:**
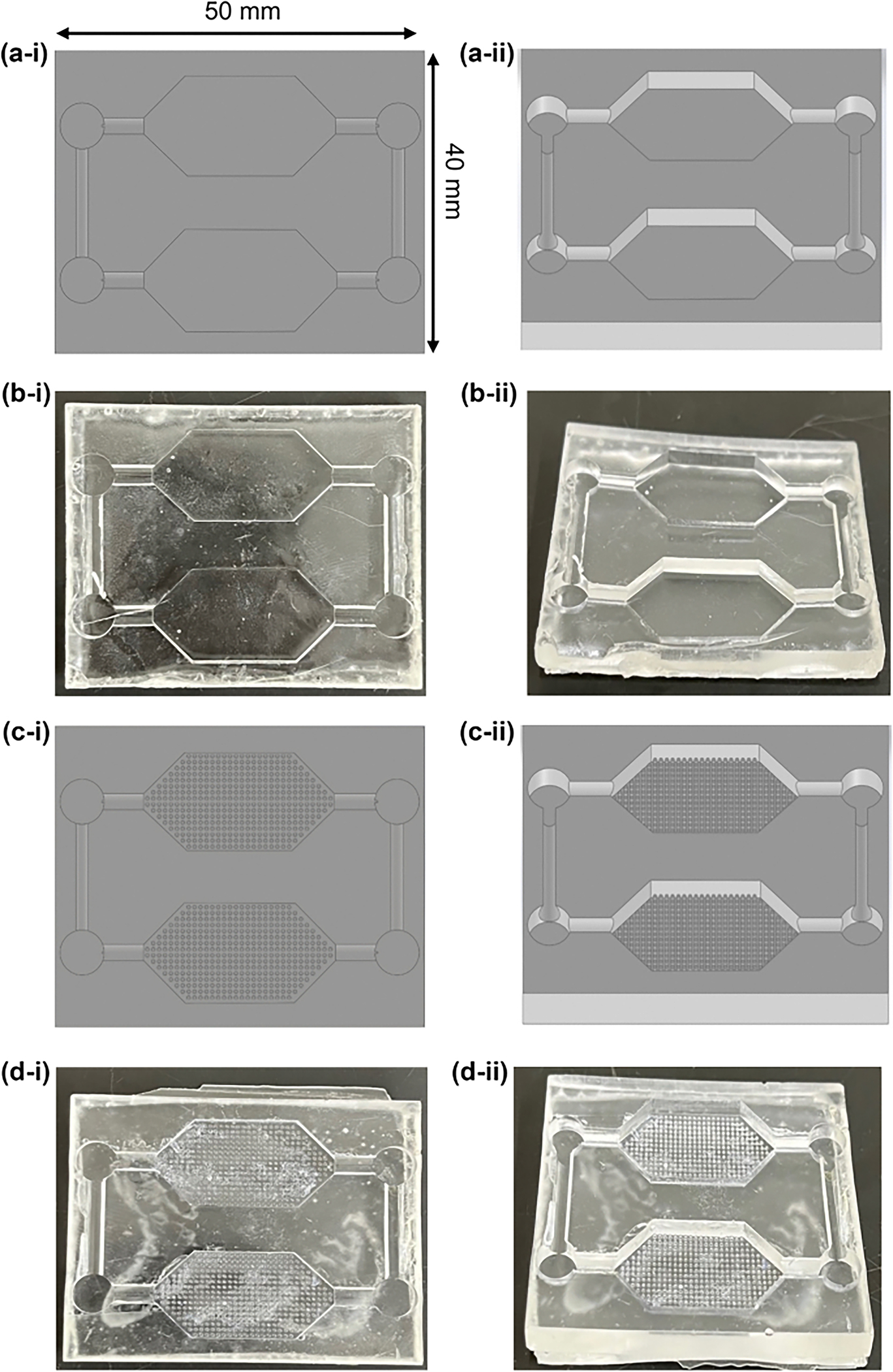
Capillary driven-flow microfluidic devices **a** Solidworks CAD device without microposts (DWoP), **b** DWoP cast with Ostemer 322, **c** Solidworks CAD device with microposts in grid arrangement within tissue culture chambers (DWPG), (**d**) DWPG cast with Ostemer

The microfluidic device designs consisted of two different main chamber designs: no microposts and grid microposts (DWoP and DWPG, respectively) (Fig. 2). The formation of the microposts were based on the design of simple capillary pumps called “tree lines” and “hexagons.” Tree lines are straight lines with equal vertical and horizontal spacing, mimicking a grid formation and referred to as DWPG. Tree lines may include microstructures, such as microposts with equal horizontal and vertical spacing that determines the capillary pressure and flow within the microfluidic device, where stronger capillary pumps have closely spaced microstructures [30]. Tree lines capillary pumps allow the main chamber to fill without trapping bubbles and microstructures (i.e. microposts) guide the liquid to continue filling the entire space [30]. The optimal dimensions of DWPG included equal spacing vertically and horizontally (0.25 mm) and diameter of hexagonal microposts (0.25 mm) maintain printing resolution of the microstructures with a Formlabs Form3B SLA printer. Hexagonal shaped microposts were integrated into the main chamber in in a grid arrangement to evaluate cell alignment and orientation in a microenvironment with biophysical stimuli (Fig. 1). Overall device dimensions include: size of microposts (0.25 mm), vertical and horizontal spacing of microposts (0.25 mm), microchannel width (2 mm), and chamber height (2.5 mm).

Based on the design considerations, capillary pressure and flow rate were numerically calculated. Capillary pressure occurs at the liquid–air interface within a microchannel as a result of surface tension of the liquid and the curvature formed by the wettable contact angle [32]. The Young–Laplace equation outlines the relationship between contact angle, microchannel size, and capillary pressure (Eq. 1) [32].

(1) $$P=-2\gamma \frac{cos\;\theta_{t}\;+\;cos\;\theta_{b}}{h}+\frac{cos\;\theta_{l}\;+\;cos\;\theta_{r}}{w}$$

where P is the capillary pressure, γ is the surface tension of the liquid, h is the channel height, w is the channel width, $\theta_{t}$ is the contact angle of the liquid with the top microchannel wall, $\theta_{b}$ is the contact angle of the liquid with the bottom microchannel wall, $\theta_{l}$ is the contact angle with the left microchannel wall, and $\theta_{r}$ is the contact angle with the right microchannel wall.

The contact angle on the microchannel walls is equal for devices built from a single material [32]. A contact angle of 60° was used for the microfluidic devices in this work based on previous studies [36]. Surface tension of water at room temperature (γ) and the height and width of the microchannels of the devices were used to calculate capillary pressure.

The Navier–Stokes equation assumes a laminar, steady state flow, and absence of gravitational effects to evaluate the flow rate (Q) of a liquid in a microchannel. The equation is as follows: [32]

(2) $$Q=\frac{h^{3}w\Delta P}{12\eta L(t)}\left[1-0.630\frac{h}{w}\right],$$

where h is the microchannel height, w is the microchannel, ΔP is the difference in capillary pressure across the microchannel, η is the fluid dynamic viscosity, and L is the length of liquid in the microchannel. The height and width of the microchannels and dynamic fluid viscosity of liquid water at room temperature were used to calculate the flow rate.

#### 2.1.2 3D stereolithography printing of microfluidic device mold

A Formlabs Form3B SLA printer with Clear V4 resin (Formlabs) was used to print the molds of the microfluidic devices. The microfluidic devices were designed in Solidworks, inverted as molds and uploaded to PreForm 3D Printing Software (Formlabs). Printing supports were added and the print job was initiated.

After printing, post-processing techniques were followed as recommended by the manufacturer for Clear V4 resin (Formlabs). Prints were removed from the build platform and submerged in the Form Wash (Formlabs) with fresh isopropyl alcohol (IPA) for 10 min [37]. The prints were air dried in the Form Wash rack. The devices were added into Form Cure (Formlabs) and UV cured at 60 °C for 15 min. After the cure, flush cutters (Formlabs) were used to carefully remove the supports from the molds [37].

#### 2.1.3 Polymer casting process to manufacture microfluidic device

The microfluidic devices were fabricated with Ostemer 322 (Mercene Labs, Sweden), which is a clear UV-curable resin [38]. Ostemer components A and B were mixed according to manufacturer specifications (A:B, 1.09:1, respectively). The device mold was cleaned with tape to remove any debris or dust and placed on a piece of aluminum foil. The Ostemer was slowly poured into the mold and air bubbles were removed with a pipette tip. The mold was placed under a UV lamp (i.e. Formlabs UV cure machine) and cured at 60 °C for 2–3 min intervals, checking for a flexible sample and allowed 3–5 min to cool down. The device was removed from the mold and placed in a furnace at 90 °C for an hour.

#### 2.1.4 Capillary fluid flow validation experiments

The microfluidic devices, DWoP and DWPG, underwent initial fluid flow experiments to determine capillary-flow. A Canon PowerShot SX620 HS camera was placed on a tripod positioned above the microfluidic device. (Supplementary Videos 1–2). The steps for adding liquid into the microfluidic device are listed below.

Trypan Blue (Fisher Scientific) and PBS (Gibco) at a ratio of 0.1 to 10, respectively were mixed thoroughly. Trypan Blue (100 μL) and PBS (10 mL) were measured into a 15 mL conical tube and mixed. A standard 1000 μL pipette tip was cut carefully using scissors to fit the inlet of the microfluidic device. The cut pipette tip was placed in the inlet of the device. Then 1 mL of the Trypan Blue and PBS mixture was released into the cut pipette in the inlet of the microfluidic device with a new, uncut pipette tip. Another 1000 μL was measured and released into the inlet. Trypan Blue and PBS were allowed to flow from the inlet to the outlet of the device, without any external aid. The experiment concluded when the outlet was completely filled. The same steps were repeated for DWPG (Supplementary Videos 1 and 2).

#### 2.1.5 Fluid flow finite element analysis (FEA)

**Fig. 3:**
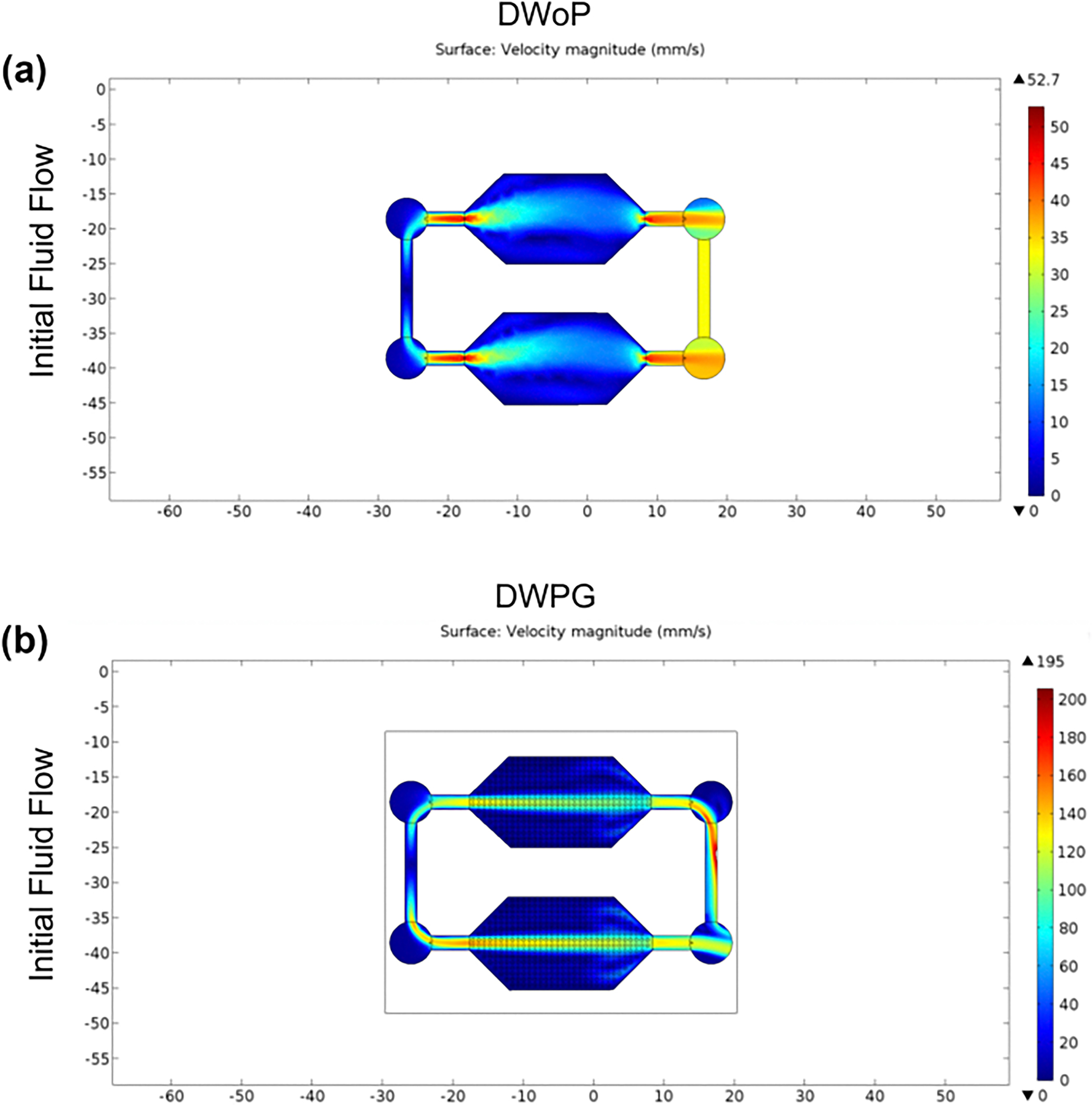
Finite Element Analysis (FEA) with COMSOL Multiphysics for DWoP (**a**) and DWPG (**b**) demonstrate closed capillary circuit fluid flow. Velocity and velocity magnitude contours based on top-left inlet location are shown

Fluid flow within the capillary circuit device was further validated with finite element analysis (FEA) using COMSOL Multiphysics software (Fig. 3). The computational simulation study parameters were modeled after experimental results from fluid flow experiments using Trypan Blue diluted in Phosphate Buffered Solution (PBS) to compare flow velocities between DWoP and DWPG in the devices made with Ostemer 322. The procedure for FEA with COMSOL Multiphysics steps simulation began with importing the 3D model of the microfluidic device and selecting stationary Laminar Flow study. Liquid water at room temperature (RT) was selected for the simulation. The inlets and outlets were added, where the inlet velocity was assigned as 1.87 mm/s and 1.39 mm/s respectively based on initial fluid flow experiments.

### 2.2 3D cardiovascular tissue model development

#### 2.2.1 Fibrin hydrogel formation

Commercially available fibrin hydrogel was used to develop the cardiovascular tissue in this study. Fibrinogen solution was prepared by dissolving 75% clottable fibrinogen (Fibrinogen from bovine plasma, Sigma Aldrich) in thrombin bovine (Thrombin, Bovine, Sigma Aldrich) in 1% bovine serum albumin (BSA, Fisher Bioreagents). Fibrinogen (25 mg) was mixed in sterile PBS warmed to 37 °C and crosslinked with thrombin after addition of cells.

#### 2.2.2 HUVEC culture and dissociation

HUVECs (ATCC, CRL-1730) between passages 4–6 were used to develop the vascular tissue model (VTM). HUVECs were cultured on 6-well plates coated with 0.2% Gelatin Type B (Sigma) with EGM-2 media (Lonza) and were passaged every 2–3 days. Once at 80% confluency, HUVECs are dissociated using Trypsin (0.25% Corning).

The trypsin was neutralized by adding Endothelial Growth Medium-2 (EGM-2, Lonza) and the cell suspension was collected and centrifuged at 1300 rpm for 3 min at 23 °C. Supernatant was aspirated carefully to not disturb the cell pellet and resuspended in 500 μL fresh EGM-2. A small amount of the cell suspension (10 μL) was removed and dispensed into a hemocytometer to count the cells with a microscope.

#### 2.2.3 AC16 culture and dissociation

AC16 human cardiomyocytes cell line (SCC109, EMD Millipore, MA) between passages 4–6 were used to develop the cardiac tissue model (CTM). AC16 were cultured in T75 flasks maintained with Dulbecco’s Modified Eagle Medium (DMEM/F12, Sigma) supplemented with 2 mM l-glutamine (EMD Millapore), 12.5% FBS (EMD Millipore), and 1 × penicillin–streptomycin solution (EMD Millipore) and passaged every 2–3 days. Once at 80% confluency, AC16s were dissociated using Trypsin (0.25% Corning). The trypsin was neutralized by adding DMEM/F12 supplemented with supplemented with 2 mM l-glutamine (EMD Millapore), 12.5% FBS (EMD Millipore), and 1 × penicillin–streptomycin solution (EMD Millipore)). Th cell suspension was centrifuged at 1300 rpm for 3 min. The supernatant was removed and the cell pellet was resuspended in 500 μL of media.

#### 2.2.4 Cell encapsulation within fibrin hydrogel

Prior to encapsulation, the thrombin in 1% BSA was prepared for crosslinking. The cells were prepared for encapsulation by following dissociation steps listed above in “HUVEC culture and dissocation” and “AC16 culture and dissocation.” The cell pellet was resuspended in 500 μL of media EGM-2 for HUVECs and DMEM/F12 for AC16s. At this step, the cells were ready for encapsulation.

With a new 1000 μL pipette tip, the cell suspension was mixed and cells were collected at densities of 500,00 cells/mL (HUVECs) and 1 × 10^6^ cells/mL (AC16s). The cells were added into the fibrinogen and PBS mixture and mixed for a homogenous mixture.

#### 2.2.5 Cell-hydrogel loading into microfluidic device

Microfluidic devices were fabricated at least 1 day prior to cell encapsulation into the fibrin hydrogel. For sterilization, microfluidic devices were placed in a glass beaker and fully immersed in 70% ethanol for 15 min. At the end of the 15 min, the microfluidic devices were allowed to completely dry before proceeding with sample loading.

When devices were sterilized and dry, 1000 μL of the cell-fibrinogen-PBS mixture was collected and slowly released into the tissue culture chambers of the microfluidic device. To crosslink the hydrogel, 150 μL of thrombin in 1% BSA was added directly to the hydrogel. The cell-hydrogel in microfluidic devices were incubated for 10–15 min at 37 °C and 5% CO_2_. The cells were supplemented with 1000 μL of media (EGM-2 for HUVECs and DMEM for AC16s) after cross linking. The microfluidic devices were placed into a 100 mm petri dish (Fisherbrand Petri Dish, 100 mm) and then into the incubator for 1, 3, and 5 days.

Transwell inserts for a multi-well plate were used as a control in this study to better assess cell morphology and behavior without exposure to Ostemer 322 and microenvironment biophysical cues (microposts). The cell-fibrinogen mixture (700 μL) was added to each Transwell and crosslinked with 150 μL of Thrombin in 1% BSA. The multi-well plate was incubated for 10–15 min at 37 °C and 5% CO_2_. After incubation, maintenance media (500 μL) was added.

For maintenance of VTM and CTM (cells encapsulated within fibrin hydrogel), spent media was removed from the microfluidic devices with a pipette tip 24 h after initial loading and supplemented with media every other day (day 3 and day 5).

### 2.3 Characterization with fluorescence microscopy and computational image analysis

#### 2.3.1 Vascular tissue model (VTM) immunofluorescence staining

On day 1, 3 and 5, HUVEC samples were rinsed with PBS (Gibco) and fixed with 4% paraformaldehyde (PFA, t ThermoFisher) for 10 min. PFA was removed and rinsed with PBS twice. Samples were permeabilized for 1 min with 0.1% Triton, rinsed two times with PBS, and blocked with 2% BSA (Bovine Serum Albumin, Fisher Bioreagents) for 30 min at 4 °C. Samples were stained with 1:200 primary anti-human CD31 (CD31 Monoclonal Antibody WM59, Invitrogen) in PBS overnight at 4 °C. The samples were rinsed twice PBS and stained with Alexa Fluor 488 goat anti-mouse secondary antibody (1:200) and Actin-stain 555 phalloidin (ActinRed 555 ReadyProbes, Invitrogen) in PBS for 1 h at room temperature (RT). Samples were rinsed twice with PBS. Right before imaging, sample nuclei were stained with DAPI (NucBlue Fixed Cell Stain ReadyProbes, Invitrogen) for 10 min at RT and washed with PBS once. Fluorescent images were captured with a Leica Thunder Imager Live Cell and 3D Assay fluorescent microscope with a 10 × objective lens (Leica Microsystems, Buffalo Grove, IL).

#### 2.3.2 Cardiac tissue model (CTM) immunofluorescence staining

On day 1, 3, and 5, AC16 samples were rinsed with PBS (Gibco) and fixed for 10 min with 4% paraformaldehyde. PFA was removed and samples rinsed using PBS twice. Samples were permeabilized for 1 min with 0.1% Triton, rinsed two times with PBS, and blocked using 2% BSA (Bovine Serum Albumin, Fisher Bioreagents) for 30 min at 4 °C. Samples were stained with 1:200 cardiac troponin T monoclonal (13–11 antibody, ThermoFisher) in PBS overnight at 4 °C. Samples rinsed twice with PBS and stained with Alexa Fluor 488 goat anti-mouse secondary antibody (1:200) and Actin-stain 555 phalloidin (ActinRed 555 ReadyProbes, Invitrogen) in PBS at 1 h at RT. Samples were rinsed twice with PBS. Right before imaging, sample nuclei were stained with DAPI (NucBlue Fixed Cell Stain ReadyProbes, Invitrogen) for 10 min at RT and rinsed with PBS once. Fluorescent images were captured with a Leica Thunder Imager Live Cell and 3D Assay fluorescent microscope with a 10 × objective (Leica Microsystems, Buffalo Grove, IL).

#### 2.3.3 Computational fluorescence image analysis—cell orientation

Cell orientation in VTM and CTM samples were quantified with ImageJ OrientationJ plugin by measuring the gradient structure in fluorescence images [39]. Images were placed in ImageJ, evaluated using Gaussian function, and exported into a histogram. Axis ranges were adjusted to include 0° to 90° preferred orientation in the images. Angles begin at 0° in the east direction and the orientation is measured counter clockwise (Supplementary Fig. 1).

#### 2.3.4 Computational fluorescence image analysis—network formation

VTM and CTM samples were evaluated for tube formation and quantified using ImageJ Angiogenesis Analyzer plugin, which measures the tube length and diameter in fluorescence images [40, 41]. The same analyzer was used to measure total network length of CTM samples to quantify cell behavior, such as cell elongation. First, the F-actin and DAPI channels (red and blue channels, respectively) were merged together to form 1 image. This image was then changed to a binary image using ImageJ and the Angiogenesis Analysis plugin measured the binary image.

##### 2.3.4.1 Statistical analysis

All quantitative measurements were performed at least in triplicate samples and values are expressed as mean ± standard deviation (SD). One- or Two-way ANOVA with post-hoc Tukey tests were used to compare treatment groups and p < 0.05 was used to assess statistical significance using GraphPad Prism Software [42].

## 3 Results

### 3.1 Capillary fluid flow validation

Validation experiments for the microfluidic devices, DWoP and DWPG, demonstrated the system’s ability to maintain capillary fluid flow in a closed circuit (Supplementary videos 1–2). The average fluid velocity was determined to be 1.87 mm/s ± 0.81 (n = 5) for DWoP and 1.39 mm/s ± 0.20 (n = 5) for DWPG. Finite element analysis with COMSOL Multiphysics further validated closed capillary circuit fluid flow within the microfluidic devices (DWoP and DWPG) (Fig. 3). At the inlet, the flow splits evenly into both channels leading into the main chamber of the devices housing the VTMs and CTMs. As flow reaches the outlets of the device, there is an increase in fluid flow allowing the fluid to continue traveling into the second chamber, indicating both are subjected to the same conditions.

### 3.2 Fluorescence microscopy

**Fig. 4:**
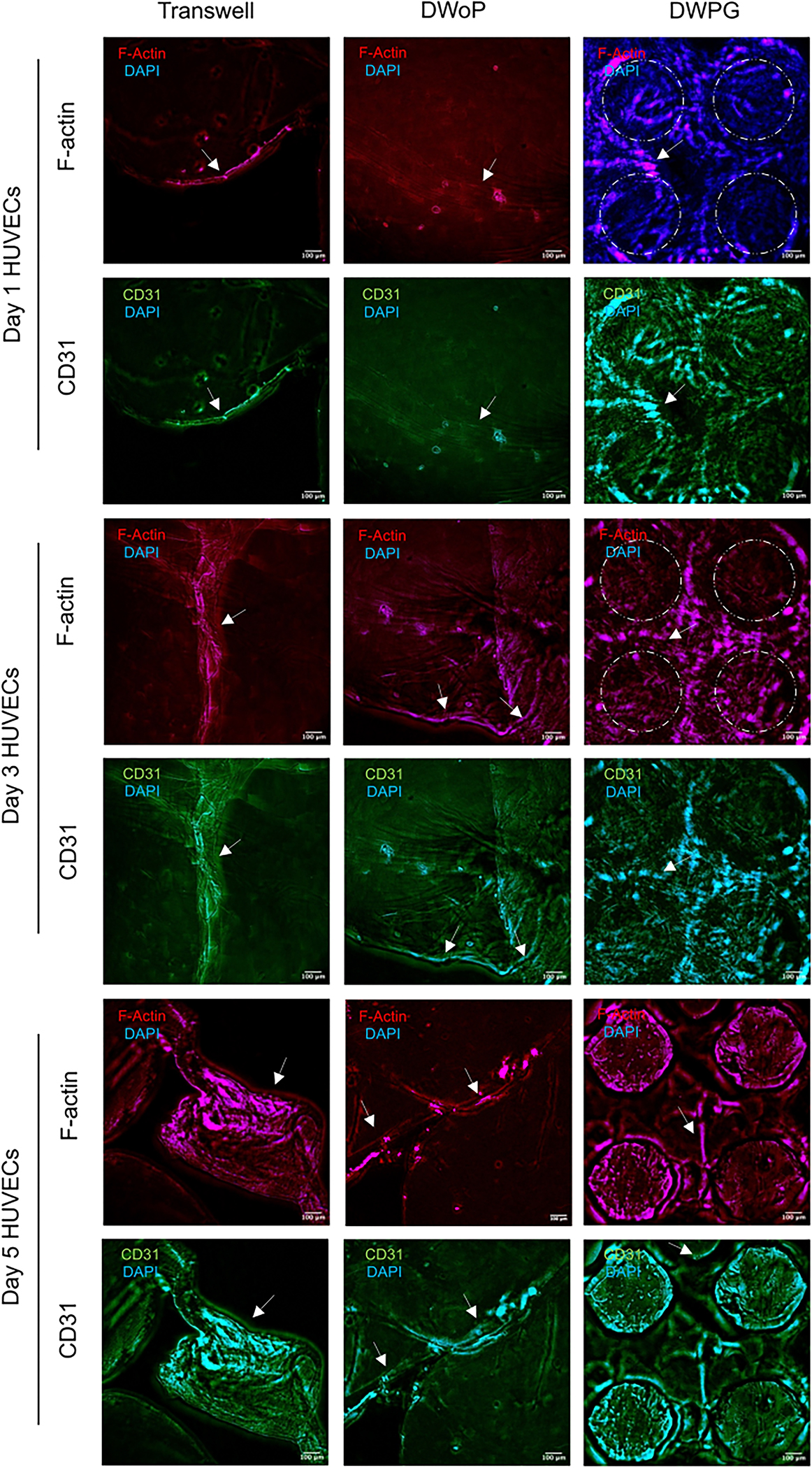
Representative 3D z-stack fluorescent images for days 1, 3, and 5 HUVECs for Transwell inserts, DWoP and DWPG. Dashed circles outline micropost locations and white arrows indicate lumen formation

Figure 4 shows fluorescence microscopy images of VTM cultured in the transwell inserts (control) and capillary circuit devices (DWoP and DWPG) for day 1, 3, and 5. The HUVECs in Transwell inserts show capillary-like tube formations at all time points. HUVECs in DWoP show capillary-like tube formations that are curved and disjointed. In DWPG, HUVECs form elongated, continuous capillary-like tubes with circular structural orientation that appears to be guided by the device microposts (dashed circles). Capillary-like lumen formation was also demonstrated in each microenvironment condition (white arrows).

**Fig. 5:**
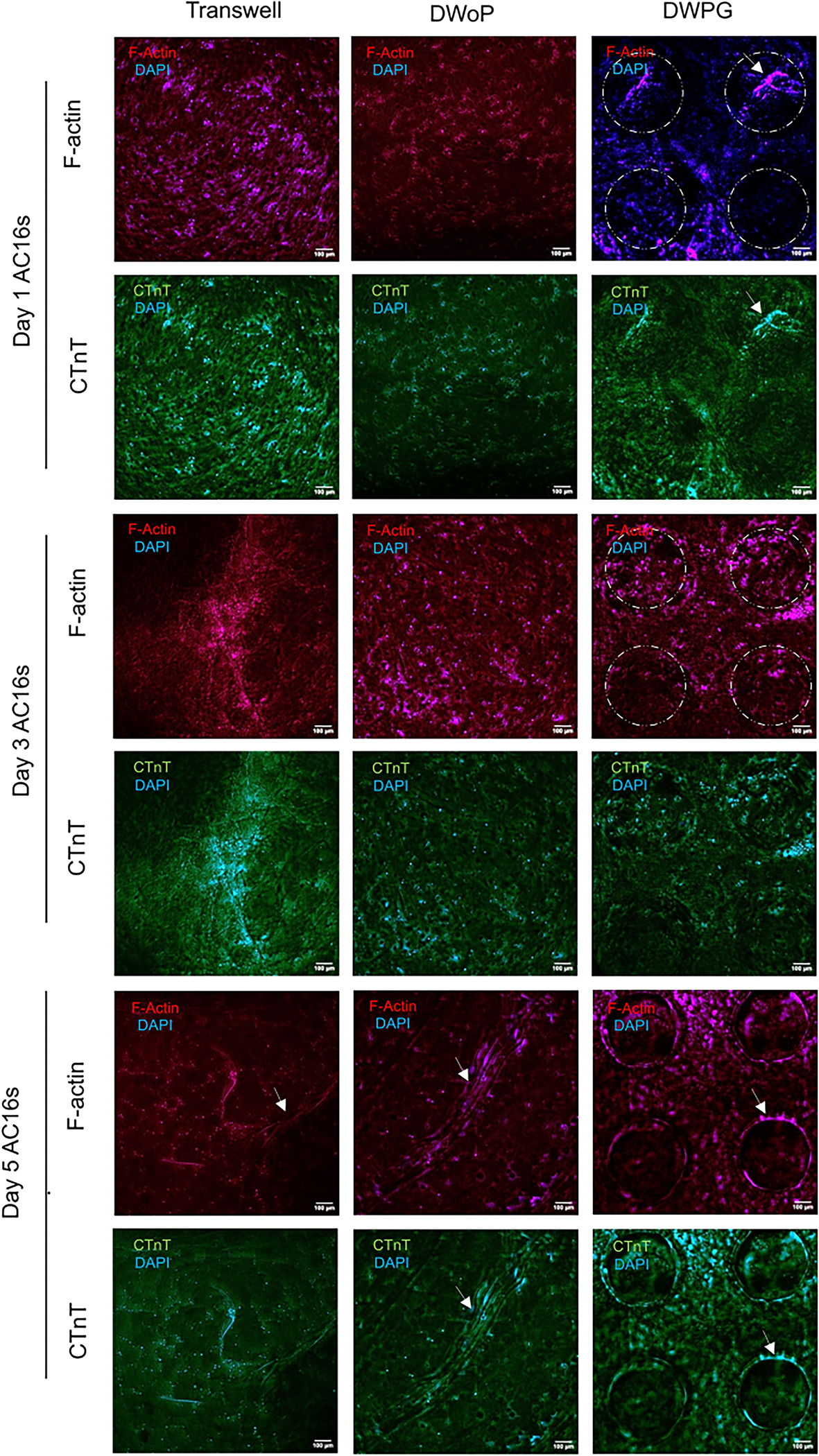
Representative 3D z-stack fluorescent images for days 1, 3, and 5 AC16s for Transwell inserts, DWoP and DWPG. Dashed circles outline micropost locations and white arrows indicate lumen formation

Figure 5 shows fluorescence microscopy images of CTM cultured in the transwell inserts (control) and capillary circuit devices (DWoP and DWPG) for day 1, 3, and 5. The AC16 cardiomyocytes in Transwell inserts show high density networks, typical in stationary 3D culture conditions. The AC16 cardiomyocytes encapsulated in DWoP show little to no alignment or specific orientation, which may be attributable to the lack of interaction between the cells and device. The AC16 cells in DWPG show alignment around the microposts, demonstrated by elongated cells arranged in circular orientations at micropost locations (dashed circles).

### 3.3 Cell orientation

**Fig. 6:**
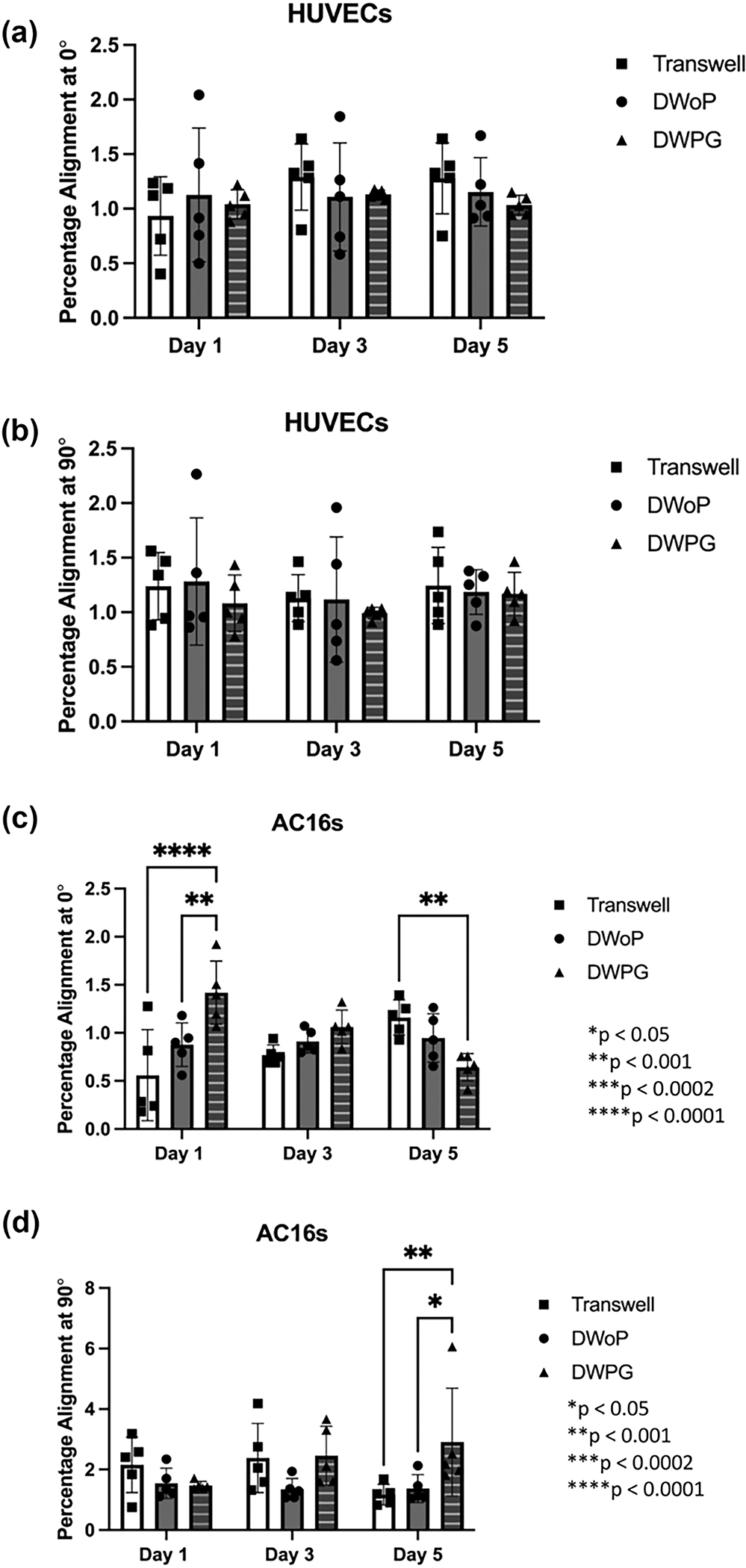
Quantification of cell percentage alignment at 0° and 90° distribution from VTM and CTM samples on day 1, 3, and 5 in Transwell, DWoP, and DWPG. (n = 5) *p < 0.05, **p < 0.002, ***p < 0.0002, ****p < 0.0001

Fluorescence images stained for F-actin cytoskeleton and nucleus visualization (Phalloidin and DAPI) from each time point and condition were analyzed using OrientationJ ImageJ plugin (Supplementary Fig. 1). Figure 6 summarizes percent cell orientation at 0° (longitudinal axis along tissue culture chamber) and 90° (transverse axis along tissue culture chamber). HUVECs in the VTMs did not demonstrate any significant differences in alignment along the 0° and 90° axis (Fig. 6A and B), this non-preferential alignment is demonstrated in the fluorescence microscopy images. In contrast, AC16 cardiomyocytes in CTMs statistically significant differences in orientation at 0° for DWPG where alignment decreased from Day 1 to Day 5 (Fig. 6C) The AC16 cardiomyocytes also demonstrated statistically significant differences in orientation at 90° for DWPG on Day 5 (Fig. 6D). These results demonstrate HUVECs are forming stable capillary-like networks with non-preferential orientation while AC16 cardiomyocyte structural organization is affected by physical cues in the microenvironment.

Overall, Day 1, 3, and 5 HUVEC Transwell samples showed different preferred orientations, ranging from 40° to 60°, whereas AC16s Transwell samples showed preferred orientation around 75° on Day 1 and by Day 3 and 5, there was a range from 40° to 80° (Supplementary Fig. 2). Day 1 HUVECs samples in DWoP, showed a preferred orientation at 60° while Day 1 AC16s samples in DWoP demonstrated preferred orientation at 80° (Supplementary Fig. 3). Day 3 and Day 5 DWoP for both cell types, did not have a preferred orientation (Supplementary Fig. 3). Day 1 and 3 HUVECs samples in DWPG did not have a preferred orientation (Supplementary Fig. 4). Day 1 AC16s DWPG did not show a preferred orientation. HUVECs Day 5 DWPG showed preferred orientation at 60°. Day 3 and 5 AC16s samples in DWPG showed preferred orientation around 90° (Supplementary Fig. 4). These results indicate cells are expanding their networks and orientations around the grid microposts.

### 3.4 Average network length

**Fig. 7:**
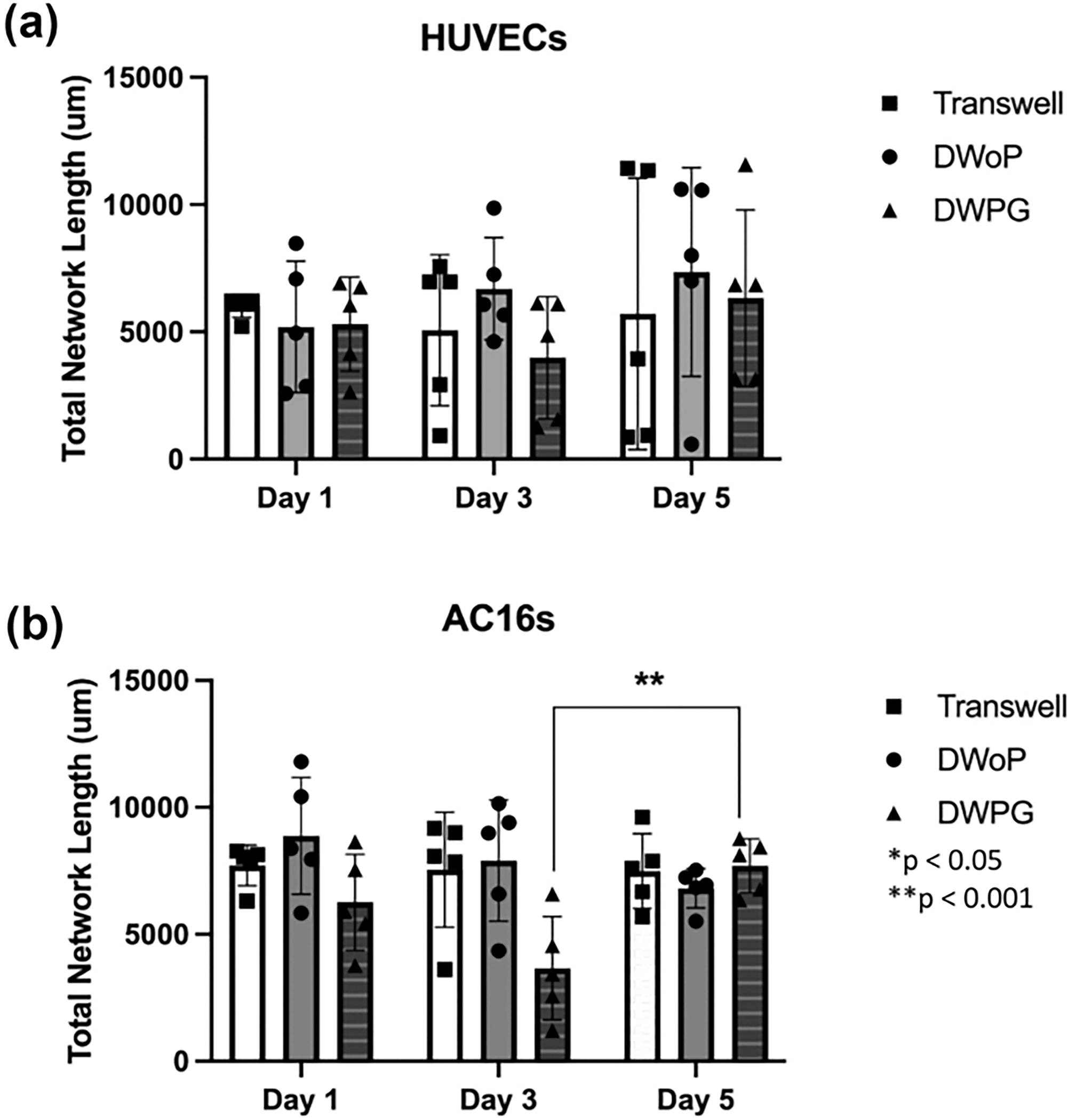
Quantification of cellular network formation within VTM (HUVECs) and CTM (AC16). Analysis of total network length in fluorescence images. **a** shows day 1, 3, and 5 data with HUVECs and **b** shows day 1, 3, and 5 data with AC16s. (n = 5) *p < 0.05, **p < 0.001

Average network length for day 1, 3, and 5 were quantified using ImageJ Angiogenesis Analyzer and the data was analyzed using GraphPad Prism. For VTM samples, there was no statistically significant difference between Transwell inserts, DWoP, and DWPG. However, for CTM samples, there was a statistical significance between Day 3 DWPG and Day 5 DWPG (Fig. 7).

## 4 Discussion

Fluorescence microscopy images and computational analysis demonstrated morphological differences between tissues cultured in DWoP vs. DWPG. In DWPG VTMs displayed capillary-like tube formation with visible cell alignment, while AC16s continued to elongate around microposts by day 5 which indicated the microposts induced biophysical cues to guide cell structure and specific organization. Computational analysis of fluorescent images resulted in statistically significant differences in alignment at 0 and 90° for CTMs and total network length. Quantifying alignment at 0° displayed a statistically significant increase in preferred alignment at 0° for Day 1 CTMs DWPG. Alignment at 0° displayed a statistically significant decrease in preferred alignment at Day 5 for CTMs in DWPG. At 90 degrees, there is a statistically significant increase in preferred alignment at 90° for Day 5 CTMs in DWPG, indicating AC16s in CTMs preferred alignment toward 90° instead of 0° after a few days in cluture. This suggests cardiomyocytes in the CTM are sensing the spacing between microposts in vertical and horizontal directions.

These results are consistent with other studies that have investigated alignment of cardiomyocytes in in vitro systems with surface microabrasions, scaffolds, ECM patterning, and microposts. Pijnappels et al. applied neonatal mesenchymal stem cells (nrMSCs), neonatal rat cardiomyocytes (nrCMCs), and neonatal rat cardio fibroblasts (nrCFs) onto channels with parallel (horizontal) or perpendicular (vertical) micro-abrasions [43]. The study demonstrated each cell type aligned according to the direction of the abrasion and during conduction testing of the tissues, cells aligned parallel to the channel displayed adjacent beating cardiac tissue. Human induced pluripotent stem cell-derived endothelial cells (iECs) and cardiomyocytes (iCMs) can be seeded into scaffolds with random or aligned microfibrous structures to analyze morphology, contractile function, and gene expression [44]. iCMs seeded in aligned scaffolds were generally more elongated and aligned following the direction of the microfibers and in iECs and iCMs coculture in aligned scaffolds, iCMs were highly aligned in the direction of the microfibers and both iECs and iCMs in random orientaed scaffolds were disorganized. Mostert et al. investigated the effect of an in vitro cococulture of human cardiomyocytes and cardio fibroblasts using extracellular matrix (ECM) protein patterning within PDMS substrates to guide the orientation (parallel, crosshatch/perpendicular, and homogenous) of both cell types [45]. Under static conditions, fluorescent images demonstrated CMs preferred orientation with parallel ECM patterning at 0° (horizontal) and coculture of CMs and CFs displayed cellular alignment for parallel patterning and no preferred orientation for crosshatch (perpendicular) patterning. Veldhuizen et al. cultured cardiac tissues in a microfluidic device with elliptical microposts and demonstrated over the course of 14 days, the proportion of cells aligned along the horizontal axis significantly increased [16]. Our results suggest microposts within the microfluidic device are providing environmental cues to help each cell type regulate their organization.

## 5 Conclusion

Our novel method and capillary-flow microfluidic device demonstrates the development of a mechanically-responsive, dynamic culture system for 3D cardiovascular tissue models with the use of stereolithography printing and polymer casting fabrication methods. Our first research questions in this study was to determine if the microfluidic device would function based on capillary-driven flow and transport liquid throughout the device without external support. Device design based on capillary-driven fluid flow and 3D printing fabrication methods resulted in a microfluidic device with a hydrophilic surface that supports self-driven flow when liquid is added to the surface. Our second research question was to determine if 3D vascular and cardiac tissues cultured within the microfluidic devices would respond to the biophysical cues from microposts and exhibit organized cell alignment. Specifically, cardiac tissue models (CTMs) in DWPG displayed preferred cell alignment toward 90° by day 5, whereas vascular tissue models (VTMs) did not show any preferred cell alignment in either device (DWPG or DWoP). This study focused on the separate culture of HUVECs and AC16s within the microfluidic device, limiting the development of a vascularized cardiac tissue. Future directions for our work and microfluidic system will include coculture studies of multiple cardiovascular cells with commercially available lines and derived from human induced pluripotent stem cell (hiPSC) for drug response studies to further develop a more physiologically-relevant, patient-specific model.

## Supplementary Material

Supplementary Figure.1. Preferred orientation is measured with OrientationJ plugin in ImageJ. The orientation is measured from z-stack fluorescence images (a) with reference to the 0 on the right side of the image (as indicated in (b)) and measured counterclockwise. Following the orientation analysis, the orientation histogram is formed (c)

Supplementary Figure 2. Representative cell orientation distribution graphs (OrientationJ ImageJ plugin) from VTM and CTM samples on day 1, 3, and 5 in Transwell inserts. (n=5)

Supplementary Figure 3. Representative cell orientation distribution graphs (OrientationJ ImageJ plugin) from VTM and CTM samples on day 1, 3, and 5 in DWoP. (n=5)

Supplementary Figure 4. Representative cell orientation distribution graphs (OrientationJ ImageJ plugin) from VTM and CTM samples on day 1, 3, and 5 in DWPG. (n=5)

Supplementary file5 (MP4 23006 kb)

Supplementary file6 (MP4 21763 kb)

**Supplementary Information** The online version contains supplementary material available at https://doi.org/10.1007/s42452-023-05459-9.

## Data Availability

The data that support the findings of this study are available from the corresponding author upon reasonable request.

## Article Highlights

- Engineered 3D human cardiac and vascular tissue models within closed circuit microfluidic device with continuous, dynamic fluid flow.
- Rapid prototyping method to fabricate closed capillary circuit microfluidic device with 3D SLA printing and polymer resin casting.
- Human vascular and cardiac cells in 3D fibrin hydrogel demonstrate network formation influenced by microfluidic device microenvironment.
